# A single-cell multi-omics atlas of human eyelid skin

**DOI:** 10.3389/fgene.2026.1780660

**Published:** 2026-03-31

**Authors:** Weiguang Ma, Yanwen Xu, Junjie Chen, Yue Yuan, Xiaoyu Wei, Qiuting Deng, Ruikang Li, Jiaxin Du, Zhongjin Zhang, Zhentao Zhou, Quhuan Li, Haiyan Shen, Jufang Zhang, Jufang Wang, Pengfei Cai, Pengcheng Guo

**Affiliations:** 1 School of Biology and Biological Engineering, South China University of Technology, Guangzhou, China; 2 BGI Research, Shenzhen, China; 3 BGI Research, Hangzhou, China; 4 State Key Laboratory of Genome and Multi-omics Technologies, BGI Research, Hangzhou, China; 5 Department of Medical Cosmetic Center, Affiliated Hangzhou First People’s Hospital, School of Medicine, Westlake University, Hangzhou, Zhejiang, China; 6 College of Life Sciences, University of Chinese Academy of Sciences, Beijing, China

**Keywords:** dataset, gene regulatory network, human skin, multi-omics, single cell

## Abstract

The skin acts as the first barrier protecting the human body against the external environment. Transcriptional heterogeneity in human skin is widely confirmed, but the regulatory mechanisms remain largely unexplored. In this study, we employed high-throughput single-cell chromatin accessibility and transcriptome sequencing (HT-scCAT-seq), a technique for simultaneous analysis of the transcriptome and epigenome. We used HT-scCAT-seq to analyze 10,065 cell profiles from four adult human eyelid skin and define 13 distinct cell types. In addition, we described detailed molecular signatures and identified key gene regulatory network enriched in each cell type. Our dataset is a valuable resource for further research in human skin biology.

## 1 Introduction

Human skin acts as a primary protective organ against the external environment. It plays key physiological roles, including protection, feel, and temperature regulation (Lefèvre-Utile et al., 2021). Skin has a complex structure with different cell types, such as keratinocytes, melanocytes, fibroblasts, and immune cells, which dynamically interact to keep tissue homeostasis and coordinate responses to environmental impacts (Fuchs, 2007; 2009). An in-depth molecular understanding of the complex interaction among these cells is key for explaining various mechanisms about skin development, aging, and disease.

Traditional bulk genomic methods have given insights into broad aspects of skin biology, but their limitation is the inability to resolve detailed molecular characterization (Polito et al., 2023). Single-cell RNA sequencing (scRNA-seq) has enhanced researchers’ capacity to identify new cell types, states, and gene regulatory pathways in human skin (Preissl et al., 2023). However, due to focusing only on gene expression, scRNA-seq provides limited insights into cellular state. Integrating single-cell data from multiple omics, such as chromatin accessibility, offers an effective method to uncover these complex regulatory networks (Stuart and Satija, 2019). Single-cell multi-omics technologies, which can measure multiple molecular aspects in the same cell at once, are essential for deepening our knowledge of skin development, homeostasis, and disease (Liu et al., 2019). The value of this multi-omics approach is the direct access to tissue samples, which allows the study of human-specific processes and disease mechanisms that may not be fully reproduced in animal models. Therefore, applying single-cell multi-omics technologies to human skin samples has enormous potential to advance our understanding of skin biology and develop new treatments for skin disease.

We generated a single-cell multi-omics map of healthy adult eyelid skin using high throughput single-cell chromatin accessibility and transcriptome sequencing (HT-scCAT-seq) (Liu et al., 2019), which concurrently detects epigenesis and transcriptome in single cells. Our dataset has collected 10,065 cells from four adults, providing a detailed molecular characterization of 13 distinct cell types in the eyelid skin. For each cell type, we profiled both chromatin accessibility and gene expression landscapes, enabling the investigation of gene regulatory mechanisms specific to cell types. Further analysis reveals key transcription factors enriched within accessible chromatin regions in each cell type, offering insights for the transcriptional networks governing cell identity and function. This resource is valuable for studying skin biology, providing a foundation for future studies investigating the molecular mechanisms underlying eyelid skin development, aging, and disease, as well as for developing targeted therapies for skin disorders.

## 2 Materials and methods

### 2.1 Sample collection and ethics

Human eyelid skin samples were collected from discarded skin tissue of eye surgeries. All procedures received ethical approval from the institutional review boards of BGI and Hangzhou First People’s Hospital. All experiments were conducted in accordance with the corresponding ethical standards.

### 2.2 Single-cell dissociation

To prepare the skin tissue, we carefully removed the macroscopic subcutaneous adipose tissue while preserving the full-thickness skin structure, including both the epidermis and the dermis. This ensured the retention of dermal appendages (such as eccrine glands and hair follicles) and the vascular network embedded within the dermis. After washing with PBS (Gibco, 10010-031), the tissue was cut into small pieces, and then digested for the preparation of single cell suspension. First, the tissue was incubated with Enzyme I (1% Penicillin/Streptomycin solution (Cytiva, SV30010) and 4 mg/mL Dispase II solution (Sigma, D4693)) under 37 °C for 50 min. After spinning down and discarding the supernatant, the tissue was incubated with Enzyme II (2.0 mg/mL collagenase type IV (BBI, A004186-0001), 1.6 mg/mL collagenase type I (BBI, A004194-0001), 18 mM CaCl2 (BBI, A600506-0100), and 1% Penicillin/Streptomycin solution (Cytiva, SV30010)) in DMEM/F-12 media under 37 °C for 30 min. The cells was passed with a 70 mm cell strainer, rinsed using 2% FBS (ExCell Bio, FSP500) in PBS, and passed again with a 30 mm cell strainer. After enrichment, the digestion solution was collected in 0.04% BSA in PBS for downstream processing.

### 2.3 Nuclei preparation and fixation

Single-nucleus suspensions for eyelid skin samples were prepared using a modified Omni-ATAC protocol (Corces et al., 2017). Briefly, cells were fixed in 0.1% formaldehyde in PBSI on ice for 5 min and quenched with 0.125 M glycine. Following fixation, cells were ruptured in cold lysis buffer (10 mM Tris-HCl pH 7.5, 10 mM NaCl, 3 mM MgCl2, 0.1% Tween-20, 0.1% NP40, 0.01% digitonin, 1% BSA in PBS, and 0.8 U/µL RNase inhibitor) on ice for 5 min, and then added with cold resuspension buffer (10 mM Tris-HCl pH 7.5, 10 mM NaCl, 3 mM MgCl2, 0.1% Tween-20, 1% BSA in PBS, and 0.8 U/µL RNase inhibitor). After centrifugation at 500 *g* under 4 °C for 5 min, nuclei were resuspended in PBS containing RNase inhibitor and SUPERase inhibitor followed by counting using DAPI staining and fixing with 0.1% formaldehyde. Fixed nuclei were added with 0.125 M glycine to neutralize the fixative, then subjected to two washes with PBSI and resuspended in PBS with RNase inhibitor.

### 2.4 Library preparation and sequencing

Fixed nuclei were subjected to *in situ* transposition using Tn5 transposase, followed by resuspension in tagmentation mix (0.08 U/µL Tn5 transposase and 1× TAG buffer in PBSI). After incubation under 37 °C for 30 min at 500 rpm, nuclei were spun down at 500 g under 4 °C for 5 min and resuspended in 8 μL of PBSI. The nuclei resuspension solution was then added to reverse transcription buffer (RTB) (0.625 mg/mL TransFlex III Reverse Transcriptase (BGI, LS-EZ-E-00027Q), 1× RT buffer, 1 mM dNTP mix, 2.5% PEG 6000, 1 mM dCTP, 3.75 µM Oligo dT, 2.5 µM TSO, and 1 U/µL RNase inhibitor). A precise temperature program was performed, increasing by 10 °C from 10 °C to 50 °C twice, to ensure reverse transcription. Following *in situ* reverse transcription, the nuclei were centrifuged at 500 *g* under 4 °C for 5 min, washed twice, and resuspended in 50 μL of 1% BSA in PBS. Libraries were prepared using the DNBelab C Series Single-Cell ATAC Library Prep Set (MGI, 940-000793-00). The protocol has been changed that a biotin-modified RNA PCR primer was added during the encapsulation and pre-amplification steps. After emulsion breakage, a 1:1 volume of MyOne C1 Dynabeads was mixed with the solution contained RNA and ATAC products in 1× B&W buffer (50 mM Tris, pH 7.5, 0.5 mM EDTA, 1 M NaCl). RNA and ATAC products were subsequently separated and processed for purification and construction. The sequencing of the libraries was performed on the DIPSEQ T1 platform at the China National GeneBank (CNGB) (Guo et al., 2020).

### 2.5 Single-cell data preprocessing

For single-cell ATAC-seq (scATAC-seq) data, the hg38 reference genome was used for reads mapping, and fragment files were generated using Chromap (Zhang et al., 2021). Barcodes were processed with d2c (v1.5.3), and peaks were identified using MACS2 to create the matrix (Zhang et al., 2008). For scRNA-seq data, reads were aligned with scStar (v1.0.3) and annotated with Anno (v1.4). One barcode was formed by the merging of beads in one droplet according to information from the scATAC-seq preprocessing. PISA (v1.10.2) was employed to create the matrix.

### 2.6 Single-cell data analysis

The gene expression and peak matrices were processed by Seurat and Signac in R (Stuart et al., 2021). We removed ambient RNA by SoupX and doublet by DoubletFinder and scDblFinder. Cells were retained based on the following criteria: number of UMI between 100 and 30,000, number of genes between 200 and 10,000, and percent.mt less than 5 for scRNA-seq; fragments more than 2000 and TSS. Enrichment score ranging from 2 to 10 for scATAC-seq data. After quality control filtering, batch effects were corrected using Harmony, and the data were normalized. For scRNA-seq data, we identified top 2000 with high variability and then applied principal component analysis for dimension reduction using ‘RunPCA’. Then we ran ‘FindNeighbors’ and ‘FindClusters’ for clustering using original Louvain algorithm in the first 15 dimensions. The cellular atlas was visualized through ‘RunUMAP’ function. Eventually, we ran ‘FindAllMarkers’ to find top differentially expressed genes. For scATAC-seq data, we used ‘RunTFIDF’ and ‘RunSVD’ functions for iterative LSI dimension reduction and then applied ‘FindClusters’ function for clustering. The clusters annotation applied the corresponding label in the same cell from the scRNA-seq data.

### 2.7 ChromVAR motif analysis

Motif activity per cell was visualized by running chromVAR with the JASPAR 2020 database (Fornes et al., 2020). Differentially enriched motifs were identified, and transcription factor (TF) motifs of interest were visualized.

### 2.8 Transcription factor footprinting analysis

Based on the motif information, we performed a TF footprinting analysis which calculates the normalized Tn5 insertion frequencies at the selected motif binding sites. The footprints were generated from aggregated “pseudobulk” signals of cell types. The footprinting results were plotted to demonstrate motif enrichment in the selected cell types (interfollicular epidermis basal cells, interfollicular epidermis spinous cells, interfollicular epidermis granular cell, proliferating keratinocyte cells, fibroblasts and macrophages) using the PlotFootprint function.

### 2.9 Transcriptional regulatory network analysis

The normalized gene expression matrix and cell-type-specific DEGs were then used as the input of GINIE3 algorithm to construct a transcriptional regulatory network linking TFs to potential target genes (Huynh-Thu et al., 2010). The TF networks were visualized using Cytoscape (Shannon et al., 2003).

### 2.10 Identification of peak-to-gene links

The peak-gene associations were identified via the LinkPeaks function, according to Pearson correlation coefficients calculated for peaks located within 50 kb of gene transcription start sites (TSSs). To mitigate the sparsity of scATAC-seq data, reads from all cells of the same cell type were aggregated to create pseudo-bulk profiles. Only peak-gene links with statistically significant positive correlations were retained.

## 3 Results

### 3.1 HT-scCAT-seq dataset of human eyelid skin

**FIGURE 1:**
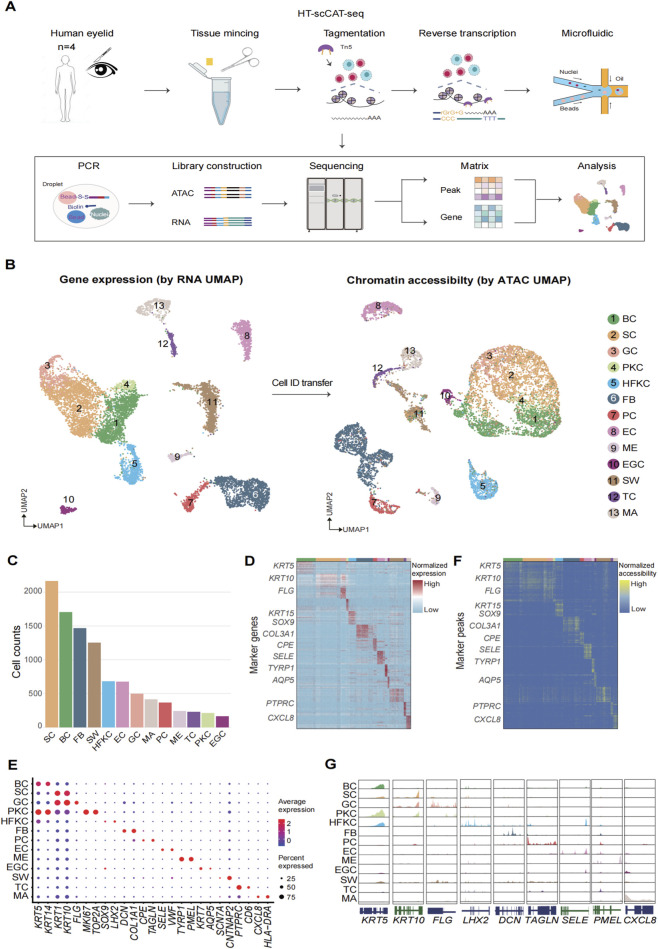
Identification of scCAT-seq data cell types of human eyelid. **(A)** A scheme of the HT-scCAT-seq experimental and data processing workflow. **(B)** UMAP projection of transcriptomic (left) and epigenomic (right) profiles from 10,065 human eyelid skin cells, with color indicating distinct cell clusters. BC, interfollicular epidermis basal cell; SC, interfollicular epidermis spinous cell; GC, interfollicular epidermis granular cell; PKC, proliferating keratinocyte cell; HFKC, hair follicle-specific keratinocytes; FB, fibroblast; PC, pericyte; EC, endothelial cell; ME, melanocyte; EGC, eccrine gland cell; SW, Schwann cell; TC, T cell; MA, macrophage. **(C)** Bar plot showing the distribution of cell counts for different cell types. **(D)** Heatmap displaying the top detected markers associated with the 13 annotated cell types. Right: the selected maker gene names. **(E)** A dot plot displaying gene expression of selected marker genes. Color bar: relative average expression levels across all clusters, dot size: percentage of cells that express the gene within each cluster. **(F)** Heatmap displaying the cell type-specific peaks. **(G)** Aggregated ATAC tracks illustrating enrichment signals in cell type-specific accessible regions.

To chart a comprehensive single-cell multi-omics atlas of human facial skin, eyelid skin specimens were collected from four adult individuals (two males, two females) undergoing routine surgical procedures. We then employed HT-scCAT-seq to characterize both the transcriptome and chromatin accessibility landscapes of individual cells after single cell isolation (Figure 1A). Briefly, the HT-scCAT-seq protocol comprises the following essential steps: 1. Nuclei isolation, permeabilization, and fixation; 2. Tn5 transposase-mediated tagging of open chromatin regions; 3. Reverse transcription and capture of mRNA transcripts using poly (dT) primers conjugated to unique molecular identifiers (UMIs); 4. Droplet-based barcoding and pre-amplification of transposed chromatin fragments and cDNA; and 5. Separation and amplification of RNA and ATAC libraries (Liu et al., 2019). Then, data processing involved libraries demultiplexing, genome alignment, barcode filtering, and generation of chromatin accessibility and gene expression matrices. Stringent quality control filters, including thresholds for several cell quality indicators, were applied to remove potential artifacts such as necrotic cells and doublets.

After quality control filtering, our dataset comprised 10,065 high-quality single-cell multi-omics profiles. For transcriptomic data, we observed a median of 2,521 UMIs and 1,834 genes per nucleus with a mitochondrial gene expression rate threshold of 5%. For chromatin accessibility, we obtained a median of 9,753 fragments per nucleus. The observed median TSS enrichment score of 4.39 is consistent with the fixation protocol employed in HT-scCAT-seq to ensure RNA integrity (Supplementary Figure S1A). Therefore, all these results demonstrate the excellent standard of our library preparation and sequencing procedures, along with the overall reliability of the resulting data.

### 3.2 HT-scCAT-seq profiling human eyelid skin cell types

To visualize and delineate the cellular composition of human eyelid skin, dimensionality reduction was performed on the RNA and ATAC data using UMAP (Figure 1B) (Becht et al., 2018). Notably, cells from all four donors were well-integrated and evenly distributed across both RNA and ATAC profiles (Supplementary Figure S1B). Referring to the established marker genes specific to cell types, we successfully identified 13 putative cell populations from the RNA-seq dataset (Figures 1B,C) (Tasic et al., 2018). These comprised interfollicular epidermis basal cells (BC, *KRT5*+), interfollicular epidermis spinous cells (SC, *KRT1*+), interfollicular epidermis granular cells (GC, *FLG*+), proliferating keratinocyte cells (PKC, *MKI67*+), hair follicle-specific keratinocytes (HFKC, *SOX9*+), fibroblasts (FB, *DCN*+), pericytes (PC, *TAGLN*+), endothelial cells (EC, *VWF*+), melanocytes (ME, *TYRP1*+), eccrine gland cells (EGC, *AQP5*+), Schwann cells (SW, *CNTNAP5*+), T cells (TC, *PTPRC*+), and macrophages (MA, *CXCL8*+) (Solé-Boldo et al., 2020; Wang et al., 2020; Zou et al., 2021) (Figures 1D,E). To integrate two omics datasets, annotated identity per cell in the scATAC-seq data was copied based on the scRNA-seq UMAP embedding (Figure 1B). Aggregated scATAC-seq signals for each identified cluster uncovered open chromatin accessibility regions near known marker gene loci (Figures 1F,G). Cells in the same cell-type annotated using scRNA-seq data exhibited highly consistent scATAC-seq profiles, further confirming their cellular identity (Supplementary Figures S1C,D).

### 3.3 Cellular heterogeneity in human eyelid skin at the gene expression and chromatin accessibility level

We proceeded to characterize each identified cell type by delineating their specific marker genes and accessible chromatin regions. The gene ontology (GO) enrichment analysis indicates known biological functions in each cell type (Supplementary Figure S1E). To establish regulatory relationships between distal chromatin elements and their cognate target genes, a peak-to-gene links analysis was performed by computing correlation for peaks located within the gene TSS, correcting for peak size and fragment counts. Peak-gene links exhibiting positive correlations were designated as putative enhancer-gene interactions (Supplementary Figure S2A) (DeTomaso and Yosef, 2021). This analysis yielded 7,514 statistically significant peak-gene linkages (correlation >0, adjusted *P* value <0.05), encompassing 6,595 regulatory elements that were linked to 1,994 cell-type-specific genes. Subsequently, we investigated whether these candidate cis-regulatory elements (cCREs) could functionally mediate the expression of differentially expressed genes (DEGs). Intriguingly, a subset of linkages involving DEGs displayed cell type-specific enrichment. For instance, a locus on chromosome 9, mapping to the promoter region of *LHX2*, exhibited the most pronounced association in HFKC (Supplementary Figure S2B). These cells also displayed elevated *LHX2* expression and heightened chromatin accessibility at this locus, suggesting that this locus represents a candidate cCRE that actively drives *LHX2* expression in HFKC. Similarly, we identified peak-gene linkages for *COL3A1*, *TAGLN*, *SELE* and *CD6* enriched in FB, PC, EC and TC.

### 3.4 Deciphering gene regulatory systems in human eyelid skin cell types

**FIGURE 2:**
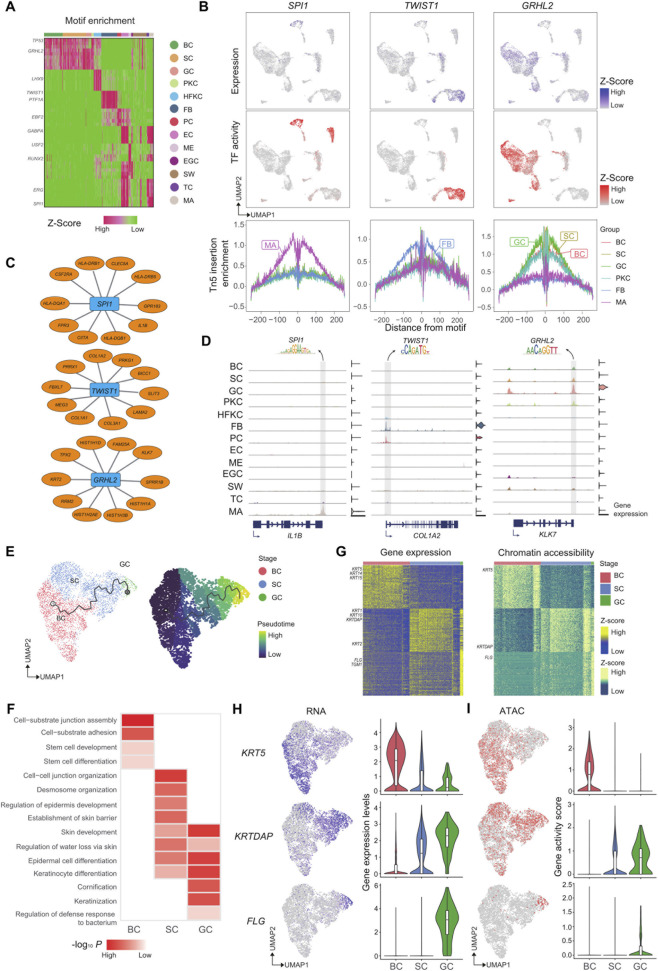
Integrated scRNA and scATAC profiles uncover celluar heterogeneity in human eyelid skin. **(A)** Heatmap displaying motif enrichment within differentially accessible chromatin regions. Right: the selected cell type-specific motif names. **(B)** Feature plots gene expression level (top) and TF activity score (middle) of underlying TFs (left: SPI1, middle: TWIST1, right: GRHL2). The line plot (bottom) shows the binding footprint of representative cell-type specific TFs. **(C)** Network visualization of predicted core regulatory TFs in 5 cell types in human skin. **(D)** Track view of aggregated ATAC signal around the *IL1B*, *COL1A2*, and *KLK7* locus. Right, violin plot shows the integrated expression levels of *IL1B*, *COL1A2*, and *KLK7* for each cell type. Gray vertical bars indicate the selected peaks related to the expression of *IL1B*, *COL1A2*, and *KLK7*, corresponding to the TF motifs SPI1, TWIST1, and GRHL2. **(E)** UMAP displaying visualizations of epidermal cell subclusters using RNA profiles and trajectory analysis. Pseudotime is depicted from dark purple to green yellow. BC, basal cell; SC, spinous cell; GC, granular cell. **(F)** Enriched GO terms for driver gene sets upregulated of the trajectory. Color reflects significance. **(G)** Heatmap showing highly expressed specifically genes (left) and peaks (right) for distinct stage. Cells are colored by type and annotated to the right. **(H)** UMAP colored by normalized gene expression level of underlying genes in epidermal cell lineage (top: *KRT5*, middle: *KRTDAP*, bottom: *FLG*). The violin plot (right) shows gene expression levels in different epidermal cell types. **(I)** UMAP colored by normalized gene activity score of underlying genes in epidermal cell lineage (top: KRT5, middle: KRTDAP, bottom: FLG). The line plot (right) shows gene activity score trends in different epidermal cell types.

To gain mechanistic insights into the regulatory underpinnings of cell type diversity, we interrogated TF motifs enriched in chromatin accessible regions (Figure 2A). This motif enrichment analysis pinpointed several potential key cell-type-specific regulators, such as SPI1 in MA, TWIST1 in FB and GRHL2 in skin epidermal cells (Figure 2B) (Chen et al., 2012; Palumbo-Zerr et al., 2017; Zhang et al., 2024). Finally, we constructed a transcriptional regulatory network to identify key TFs controlling gene expression in each cell type (Figure 2C). This network provides a comprehensive overview of the regulatory landscape governing cell identity and function in human eyelid skin. Intriguingly, the predicted target genes of these TFs, such as *IL1B* for SPI1, *COL1A2* for TWIST1, and *KLK7* for GRHL2, exhibited strong functional relevance to their corresponding cell types (Figure 2D).

### 3.5 Integrated multi-omics analysis reveals synchronized epigenetic and transcriptional programs driving epidermal differentiation

To delineate the differentiation dynamics within the epidermis, we performed a pseudotime trajectory analysis using Monocle 3 based on the high-resolution scRNA-seq data. This analysis successfully reconstructed a continuous differentiation trajectory of keratinocytes, originating from basal cells, progressing through a spinous keratinocyte intermediate state, and culminating in terminally differentiated granular cells (Figure 2E). GO enrichment analysis along this trajectory revealed a clear functional progression that mirrors the known biology of epidermal stratification (Figure 2F). Consistent with their progenitor function, basal cells at the start of the trajectory were significantly enriched for genes associated with stem cell development and differentiation. As cells moved along the pseudotime axis, spinous keratinocytes showed a strong enrichment for processes integral to building the epidermal layers, including epidermis development, epidermal cell differentiation, and keratinocyte differentiation. At the trajectory’s endpoint, the granular cell population was characterized by the upregulation of genes involved in keratinization and regulation of defense response to bacterium, highlighting their critical roles in forming the skin barrier and contributing to innate immunity. Leveraging the paired nature of our multi-omics dataset, we projected the scATAC-seq chromatin accessibility profiles onto this RNA-inferred backbone to interrogate the relationship between the epigenetic landscape and transcriptional output. By performing a dynamic enrichment analysis along the pseudotime axis, we observed a striking concordance between gene expression levels and chromatin accessibility at lineage-specifying loci (Figure 2G). The distinct accessibility peaks within the promoters and distal regulatory elements of key differentiation markers (e.g., *KRT5*, *KRTDAP*, *FLG*) showed a progressive increase in openness that paralleled their transcriptional induction (Figures 2H,I). This high degree of coupling suggests that the differentiation of basal cells into spinous and granular layers is driven by a tightly coordinated regulatory mechanism, where chromatin remodeling events either prime or instantaneously license the transcription of differentiation-specific programs. These results underscore a deterministic link between the establishment of an accessible chromatin landscape and the execution of the epidermal terminal differentiation program.

## 4 Conclusion

This study presents a comprehensive single-cell multi-omics atlas of adult human eyelid skin, generated using HT-scCAT-seq. By simultaneously profiling the transcriptome and chromatin accessibility in 10,065 cells, we have defined 13 distinct cell types and provided a detailed characterization of their molecular signatures. Our integrated analysis reveals a high degree of coordination between epigenetic landscapes and transcriptional programs, uncovering cell-type-specific gene regulatory networks and identifying key transcription factors that underpin cellular identity and function. Furthermore, trajectory analysis delineated the synchronized epigenetic and transcriptional dynamics that drive epidermal differentiation.

This study provides a comprehensive dataset that illuminates the cis-regulatory logic governing cell fate specification and differentiation in human eyelid skin. Further in-depth exploration of this resource will undoubtedly advance our understanding of human skin aging and disease mechanisms, potentially paving the way for the development of novel strategies for disease prevention and cell-based therapeutic interventions.

## Data Availability

The original contributions presented in the study are publicly available. All raw sequencing data are deposited under accession number CNP0006705 in CNGB Nucleotide Sequence Archive (CNSA; https://db.cngb.org/cnsa/).

## Supplementary material

SUPPLEMENTARY FIGURE S1 Quality control of scCAT-seq data, UMAP plot and GO cell types **(A)** Box plot illustrating the distribution of UMI, gene, fragments and TSS enrichment score for each library. **(B)** UMAP projection of transcriptomic (top) and epigenomic (bottom) profiles from four donors colored by donor ID (right). Relative expression of gene **(C)** and chromatin accessibility **(D)** characteristic for BC, SC, GC and FB. **(E)** Enriched Gene Ontology (GO) Terms for cell types in Figure 1B.

SUPPLEMENTARY FIGURE S2 Peak-to-gene links uncovered heterogeneity of human eyelid skin. **(A)** Heatmap showing gene expression (left) and chromatin accessibility (right) for 7,514 significantly linked CRE-gene pairs. Each row represents a linked gene and a pair of CREs. Bar on the top represents the cell types involved in skin. **(B)** Track view of aggregated ATAC signal around the *LHX2, COL3A1, TAGLN, SELE, CD6* locus. Peaks and peak-to-gene linkages are shown below the tracks. Right, violin plot shows the integrated expression levels of *LHX2, COL3A1, TAGLN, SELE, CD6* for each cell type.
