# Supplementary material for: A single-cell multi-omics atlas of human eyelid skin
Source: Front Genet. 2026 Mar 31;17:1780660. doi: 10.3389/fgene.2026.1780660 (PMC13075857; doi:10.3389/fgene.2026.1780660)
Supplement: Supplementary file 1 [file Supplementaryfile1.docx]

Supplementary Material

# Supplementary Figures

**Supplementary Figure 1. Quality control of scCAT-seq data, UMAP plot and GO cell types**

**(A)** Box plot illustrating the distribution of UMI, gene, fragments and TSS enrichment score for each library. **(B)** UMAP projection of transcriptomic (top) and epigenomic (bottom) profiles from four donors colored by donor ID (right). Relative expression of gene  **(C)** and chromatin accessibility **(D)** characteristic for BC, SC, GC and FB. **(E)** Enriched Gene Ontology (GO) Terms for cell types in Figure 1B.

**Supplementary Figure 2. Peak-to-gene links uncovered heterogeneity of human eyelid skin.**

**(A)** Heatmap showing gene expression (left) and chromatin accessibility (right) for 7,514 significantly linked CRE-gene pairs. Each row represents a linked gene and a pair of CREs. Bar on the top represents the cell types involved in skin. **(B)**Track view of aggregated ATAC signal around the *LHX2, COL3A1, TAGLN, SELE, CD6* locus. Peaks and peak-to-gene linkages are shown below the tracks. Right, violin plot shows the integrated expression levels of *LHX2, COL3A1, TAGLN, SELE, CD6* for each cell type.
