# Supplementary figures and images for: A single-cell multi-omics atlas of human eyelid skin
Source: Front Genet. 2026 Mar 31;17:1780660. doi: 10.3389/fgene.2026.1780660 (PMC13075857; doi:10.3389/fgene.2026.1780660)

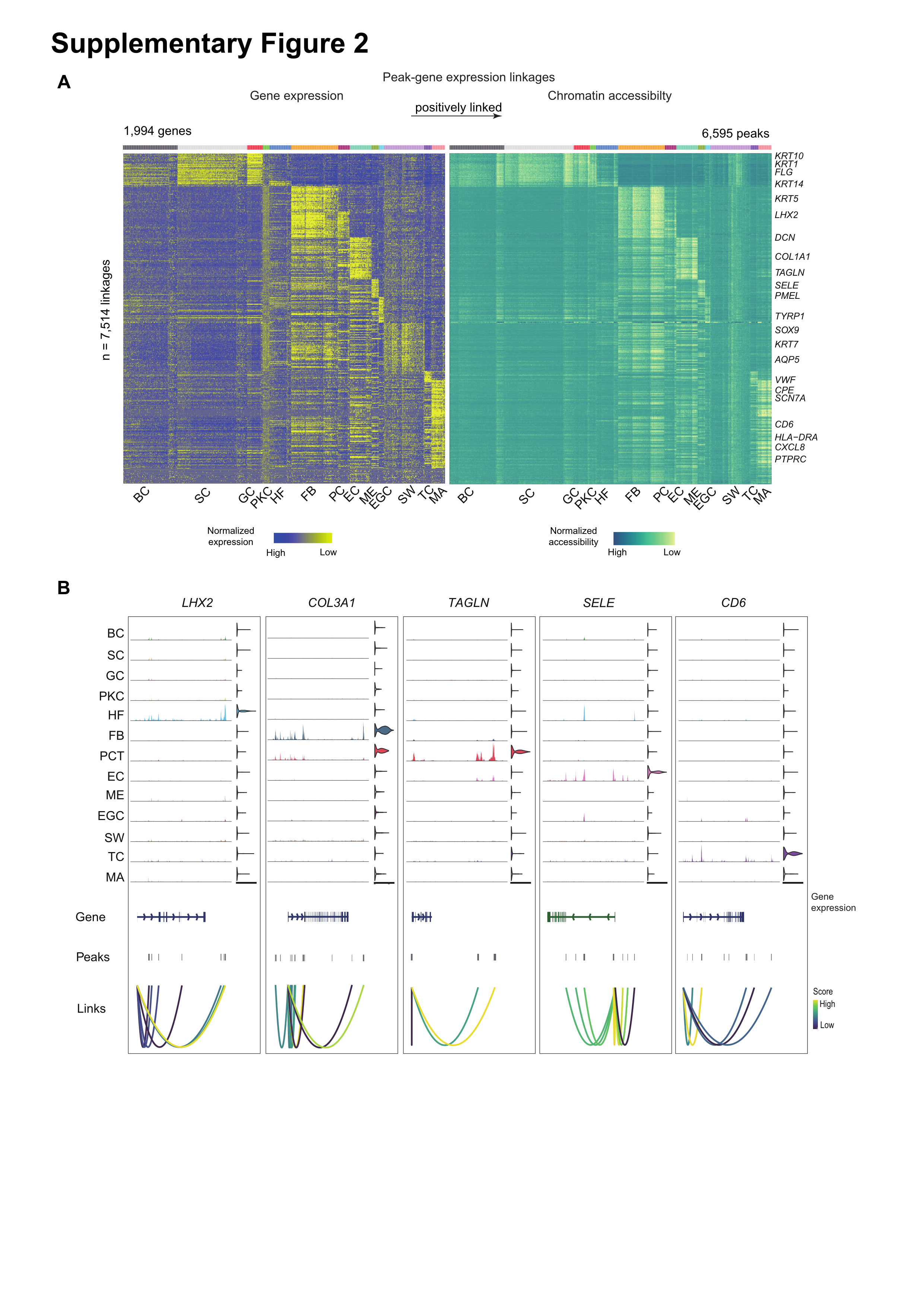

Supplement: Supplementary file 2 [file Image2.tif]

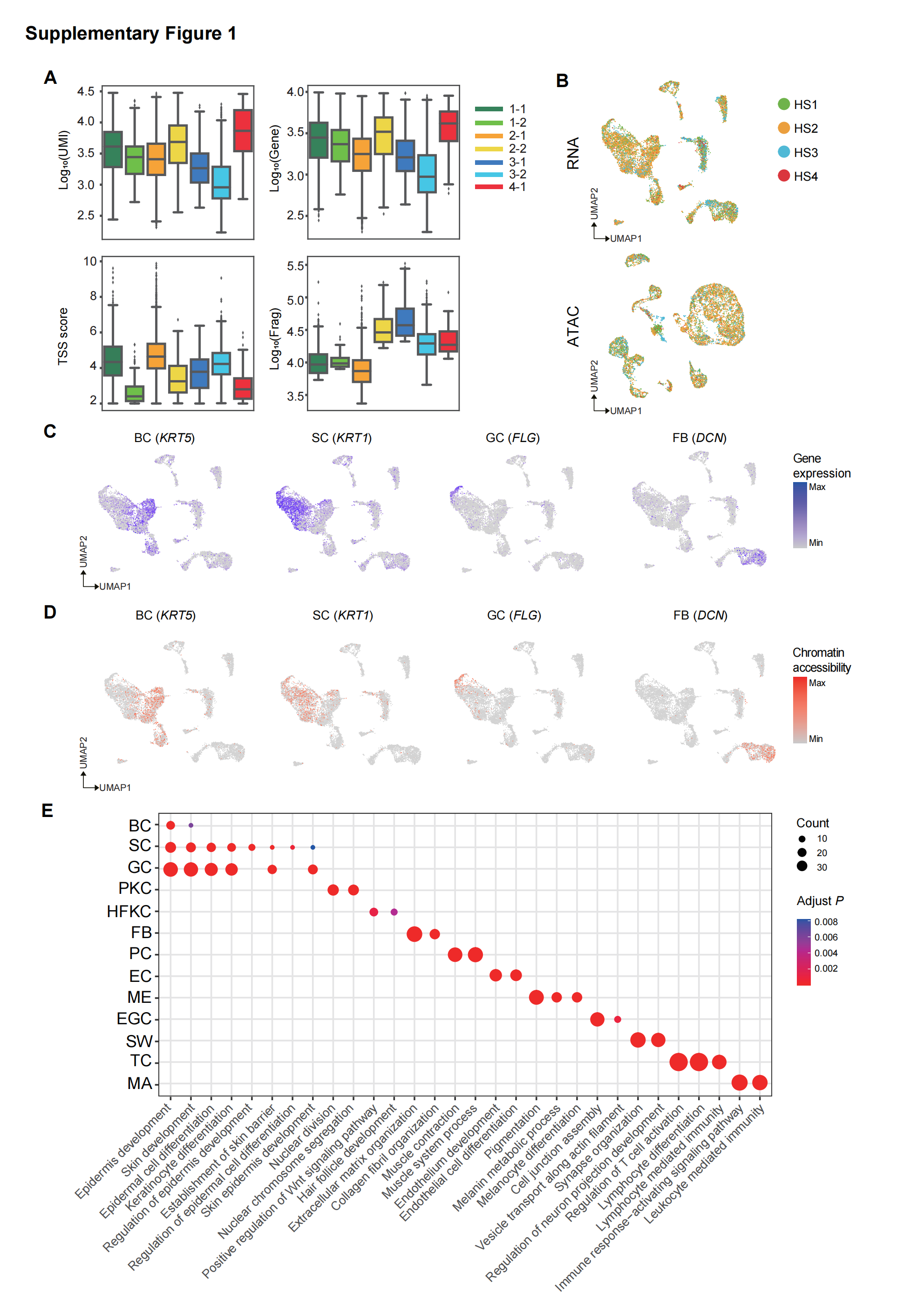

Supplement: Supplementary file 3 [file Image1.tif]
